# Cardiovascular disease (CVD) and chronic kidney disease (CKD) event rates in HIV-positive persons at high predicted CVD and CKD risk: A prospective analysis of the D:A:D observational study

**DOI:** 10.1371/journal.pmed.1002424

**Published:** 2017-11-07

**Authors:** Mark A. Boyd, Amanda Mocroft, Lene Ryom, Antonella d’Arminio Monforte, Caroline Sabin, Wafaa M. El-Sadr, Camilla Ingrid Hatleberg, Stephane De Wit, Rainer Weber, Eric Fontas, Andrew Phillips, Fabrice Bonnet, Peter Reiss, Jens Lundgren, Matthew Law

**Affiliations:** 1 Kirby Institute, University of New South Wales, Sydney, Australia; 2 Faculty of Health and Medical Sciences, University of Adelaide, Adelaide, South Australia, Australia; 3 Department of Infection and Population Health, University College London, London, United Kingdom; 4 Centre of Excellence for Health, Immunity and Infections, Department of Infectious Diseases, Rigshospitalet, University of Copenhagen, Copenhagen, Denmark; 5 Dipartimento di Scienze della Salute, Clinica di Malattie Infettive e Tropicali, Azienda Ospedaliera–Polo Universitario San Paolo, Milan, Italy; 6 ICAP at Columbia University, New York, New York, United States of America; 7 Division of Infectious Diseases, Saint-Pierre University Hospital, Université Libre de Bruxelles, Brussels, Belgium; 8 Department of Infectious Diseases and Hospital Epidemiology, University Hospital Zurich, University of Zurich, Zurich, Switzerland; 9 Department of Public Health, Nice University Hospital, Nice, France; 10 Centre Hospitalier Universitaire de Bordeaux, Université de Bordeaux, Bordeaux, France; 11 Bordeaux Population Health, INSERM U1219, Université de Bordeaux, Bordeaux, France; 12 Department of Global Health, Academic Medical Center, University of Amsterdam, Amsterdam, The Netherlands; 13 Division of Infectious Diseases, Academic Medical Center, University of Amsterdam, Amsterdam, The Netherlands; 14 HIV Monitoring Foundation, Amsterdam, The Netherlands; San Francisco General Hospital, UNITED STATES

## Abstract

**Background:**

The Data Collection on Adverse Events of Anti-HIV Drugs (D:A:D) study has developed predictive risk scores for cardiovascular disease (CVD) and chronic kidney disease (CKD, defined as confirmed estimated glomerular filtration rate [eGFR] ≤ 60 ml/min/1.73 m^2^) events in HIV-positive people. We hypothesized that participants in D:A:D at high (>5%) predicted risk for both CVD and CKD would be at even greater risk for CVD and CKD events.

**Methods and findings:**

We included all participants with complete risk factor (covariate) data, baseline eGFR > 60 ml/min/1.73 m^2^, and a confirmed (>3 months apart) eGFR < 60 ml/min/1.73 m^2^ thereafter to calculate CVD and CKD risk scores. We calculated CVD and CKD event rates by predicted 5-year CVD and CKD risk groups (≤1%, >1%–5%, >5%) and fitted Poisson models to assess whether CVD and CKD risk group effects were multiplicative. A total of 27,215 participants contributed 202,034 person-years of follow-up: 74% male, median (IQR) age 42 (36, 49) years, median (IQR) baseline year of follow-up 2005 (2004, 2008). D:A:D risk equations predicted 3,560 (13.1%) participants at high CVD risk, 4,996 (18.4%) participants at high CKD risk, and 1,585 (5.8%) participants at both high CKD and high CVD risk. CVD and CKD event rates by predicted risk group were multiplicative. Participants at high CVD risk had a 5.63-fold (95% CI 4.47, 7.09, *p <* 0.001) increase in CKD events compared to those at low risk; participants at high CKD risk had a 1.31-fold (95% CI 1.09, 1.56, *p* = 0.005) increase in CVD events compared to those at low risk. Participants’ CVD and CKD risk groups had multiplicative predictive effects, with no evidence of an interaction (*p* = 0.329 and *p* = 0.291 for CKD and CVD, respectively). The main study limitation is the difference in the ascertainment of the clinically defined CVD endpoints and the laboratory-defined CKD endpoints.

**Conclusions:**

We found that people at high predicted risk for both CVD and CKD have substantially greater risks for both CVD and CKD events compared with those at low predicted risk for both outcomes, and compared to those at high predicted risk for only CVD or CKD events. This suggests that CVD and CKD risk in HIV-positive persons should be assessed together. The results further encourage clinicians to prioritise addressing modifiable risks for CVD and CKD in HIV-positive people.

## Abstract 2

In an observational study, Mark Boyd and colleagues report on risks of cardiovascular and renal events in people with HIV infection.

## Author summary

**Why was this study done?:**

- Access to combination antiretroviral therapy has increased the life expectancy of HIV-positive people.
- Despite this success, there is evidence that HIV-positive people may experience an earlier onset of comorbidities (e.g., cardiovascular disease [CVD] and chronic kidney disease [CKD]) in comparison with their HIV-negative peers.
- The D:A:D study has developed specific models to predict the risk for CVD and CKD events in HIV-positive people.
- This study was designed to investigate whether HIV-positive people at high risk for both CVD and CKD are at even greater risk for CVD and CKD events, and may therefore warrant particularly close clinical attention and management.

**What did the researchers do and find?:**

- The D:A:D study is a prospective observational study that combines 11 cohorts of >49,000 HIV-positive people followed prospectively since 1999.
- We performed a study that included the 27,215 participants with the requisite data to calculate their D:A:D CVD and CKD risk scores. We calculated the CVD and CKD event rates by predicted 5-year CVD and CKD risk groups (≤1%, >1%–5%, >5%) and analysed whether CVD and CKD risk group effects were multiplicative.
- We found that people at high predicted risk for both CVD (>5%) and CKD (>5%) events have substantially greater risks for both CVD and CKD events compared with those at low predicted risk for both outcomes and those at high predicted risk for CVD or CKD events alone.

**What do these findings mean?:**

- HIV-positive people not uncommonly have high predicted CVD and/or CKD risk; these interact to create substantial risks for future morbid events.
- Our results suggest that CVD and CKD risk in HIV-positive people should be assessed together.
- The results should further encourage clinicians to prioritise addressing modifiable risks for CVD and CKD in HIV-positive people.

## Introduction

Combination antiretroviral therapy has transformed the lives of HIV-positive people. Over the past 20 years in high-income countries, rates of opportunistic diseases have declined and life expectancy has reached levels similar to that of the HIV-negative population [1–4]. However, there is evidence suggesting that people living with HIV experience greater and earlier onset of comorbidities compared with their HIV-negative peers [5–7]. The relative extent to which this observation relates to a higher prevalence of behaviours and risks associated with such comorbidities, increased levels of immune activation and coagulopathy despite sustained virological suppression, antiretroviral treatment, and/or other mechanisms remains unclear [8,9].

In the general population, it is well established that chronic kidney disease (CKD) is an important and independent risk factor (covariate) for cardiovascular disease (CVD) [10]. It has been argued that CKD should be considered a coronary risk factor, and that aggressive risk factor reduction for other CVD risk factors should be part of standard therapy for patients with CKD [11].

CVD in turn is associated with CKD. This may be mediated by the development of atherosclerosis, or may result as a consequence of shared risk factors with CKD such as hypertension and diabetes mellitus. It is well recognised that the effects of comorbidity may be greater than the effects of the sum of risk of each disease, and that their co-existence may lead to more severe illness, poorer prognosis, and premature death [11].

In HIV-positive people, a similarly strong relationship between CKD and CVD has been reported [12]. The Data Collection on Adverse Events of Anti-HIV Drugs (D:A:D) collaboration has developed HIV-specific predictive risk models for CVD [13] and CKD [14] in an attempt to help guide care in these individuals. We hypothesized that participants in D:A:D at high (>5%) predicted risk for both CVD and CKD would be at even greater risk for CVD and CKD events, and may therefore warrant particularly close clinical attention and management.

## Methods

The D:A:D study is a prospective observational study that combines 11 cohorts of HIV-positive people. Its objective is to establish whether the use of combination antiretroviral therapy is associated with an elevated risk of CVD, end-stage renal disease (ESRD), liver disease, cancer, and death. The 11 cohorts contribute data on 49,717 participants enrolled at 212 clinics in Europe, the US, Argentina, and Australia. The standardised dataset includes information on socio-demographics, AIDS events and death, known CVD risk factors, height and weight, laboratory markers, antiretroviral treatment history, estimated glomerular filtration rate (eGFR) (Cockcroft—Gault equation), and treatments including those prescribed for CVD risk.

The main analyses in this study were performed according to a prospectively established statistical analysis plan (S2 Text). The non-prospectively defined analyses were those investigating the covariates of the CVD risk score as predictors for CKD events adjusted for CKD risk group, the covariates of the CKD risk score as predictors for CVD events adjusted for CVD risk group, and the prevalence of diabetes at baseline according to predicted CKD and CVD risk group.

All participating cohorts in the D:A:D study followed local national guidelines/regulations regarding patient consent and ethical review.

### Participants

In this study we included individuals for whom a complete set of risk covariate data was available after 1 January 2004 as required by both the D:A:D CVD and CKD risk equations, except for missing data for exposure to viral hepatitis C and family history of CVD, in which case these were imputed to be negative (thus slightly underestimating CVD risk in these individuals). All those included in the analysis had an eGFR > 60 ml/min/1.73 m^2^ at baseline and an eGFR ≤ 60 ml/min/1.73 m^2^ observed subsequently on at least 2 consecutive occasions at least 3 months apart. We used definitions of endpoints indicating CVD and CKD as defined in previous D:A:D publications [13,14], but note that CVD events were centrally validated clinical events (myocardial infarction, stroke, and invasive cardiac procedures), whereas CKD events were based on confirmed laboratory criteria.

Patient follow-up was censored at the earliest of the following: last eGFR, last visit plus 6 months, or 1 February 2015. We excluded participants with a previous history of CVD, baseline eGFR ≤ 60 ml/min/1.73 m^2^, or a CVD or CKD event that occurred within 30 days of baseline. We included CVD events and follow-up after the definition of a CKD event had been met and CKD events after the definition of a CVD event had been met.

We calculated the 5-year predicted risk for CKD and CVD using the D:A:D risk equations [13,14]. The CVD equation includes age, sex, diabetes, CVD family history, current and former smoking, total cholesterol, high-density lipoprotein (HDL) cholesterol, systolic blood pressure, current CD4 count, current receipt of abacavir, and cumulative exposure to protease inhibitors (PIs) and nucleoside/nucleotide reverse transcriptase inhibitors (N[t]RTIs). We censored PI and N(t)RTI at 3 and 7 years, respectively, as this reflects the range of data on which the models were developed and so avoids over-predicting risk. The CKD equation includes age, sex, exposure to HIV through injecting drug use, hepatitis C coinfection, baseline eGFR, nadir CD4 count, hypertension, prior CVD and diabetes, adjustment for current receipt of unboosted atazanavir or ritonavir-boosted lopinavir, and a higher adjustment for tenofovir disoproxil fumarate, ritonavir-boosted atazanavir and any other PI.

We stratified the 5-year predicted CKD and CVD risk into groups as ≤1%, >1%–5%, and >5%. We did this rather than use predicted risk as a continuous measure for 2 reasons. First, this reflects how clinicians and their patients use these risk equations, categorising predicted risk into groups that then indicate possible interventions. Second, using risk groups in this fashion allows clear presentation of the rates of CKD and CVD events in each predicted risk group. We deliberately chose not to use the low, moderate, and high CKD risk groups defined by Mocroft et al. [14] in order that the CVD and CKD risk groups could be interpreted in the same fashion. We performed a secondary analysis of events using the Framingham equation, recalibrated to the D:A:D study as described in a previous publication [13], to assess whether the results would be similar compared to the primary analysis. We also examined CKD and CVD events using predicted risks as continuous covariates as a sensitivity analysis.

### Statistical methods

We calculated CKD and CVD event rates by CKD and CVD risk group. We fitted Poisson models to assess whether CKD and CVD risk group effects are additive or multiplicative. In an attempt to assess how the predicted CVD risk score was contributing to prediction of CKD events, we investigated predictors of CKD events, fitting the CKD risk score group and then each of the covariates in the CVD risk score that are not included in the CKD risk score. We also investigated predictors of CVD events, fitting the CVD risk score group and then each of the covariates in the CKD risk score that are not included in the CVD risk score.

## Results

### Baseline characteristics

Of the 49,717 individuals enrolled in the D:A:D study, 27,215 (55%) had the required complete covariate data after 1 January 2004 and were included in this analysis, contributing 202,034 person-years (pyrs) of follow-up.

Characteristics of the 27,215 individuals included at baseline, defined as the first time each individual had complete CVD and CKD risk factor data available after 1 January 2004, are shown in Table 1. The median (IQR) baseline year of follow-up was 2005 (2004, 2008). The cohort was 74% male, with a median (IQR) age of 42 (36, 49) years. Fifty percent were smokers at baseline, and 3.8% had a diagnosis of diabetes mellitus. The median (IQR) 5-year predicted CKD risk was 1.1% (0.6%, 3.7%), and the median (IQR) 5-year predicted CVD risk was 1.6% (0.8%, 3.3%).

**Table 1:** Participant baseline characteristics.

Characteristic	*N* (%) or median (IQR)
Overall study population	27,215 (100%)
Male	20,206 (74.3%)
IDU exposure to HIV	3,673 (13.5%)
Current smoker	13,466 (49.5%)
Ex-smoker	5,466 (20.1%)
Diabetes	1,031 (3.8%)
Family history of CVD	2,257 (8.3%)
HCV positive^1^	5,276 (19.4%)
HBV positive^2^	1,462 (5.4%)
Receiving abacavir	4,551 (16.7%)
Receiving tenofovir	8,212 (30.2%)
Receiving atazanavir	2,336 (8.6%)
Receiving indinavir	559 (2.1%)
Receiving lopinavir	4,522 (16.6%)
Receiving ritonavir	8,295 (30.5%)
Age (years)	42 (36, 49)
eGFR (ml/min/1.73 m^2^)	100 (86, 117)
Total cholesterol (mmol/l)	4.8 (4.1, 5.7)
HDL cholesterol (mmol/l)	1.2 (0.9, 1.5)
CD4 count (cells/mm^3^)	464 (319, 650)
Systolic BP (mm Hg)	120 (113, 130)
Diastolic BP (mm Hg)	80 (70, 82)
Cumulative PI use (years)	0.9 (0, 4.0)
Cumulative N(t)RTI use (years)	3.9 (0, 4.0)
5-year predicted CKD risk	1.1% (0.6%, 3.7%)
5-year predicted CVD risk	1.6% (0.8%, 3.3%)
Year of baseline	2005 (2004, 2008)

^1^Defined as HCV antibody or HCV RNA positive.

^2^Defined as viral hepatitis B surface antigen positive, e antigen positive, or HBV DNA positive.

BP, blood pressure; CKD, chronic kidney disease; CVD, cardiovascular disease; eGFR, estimated glomerular filtration rate; HBV, hepatitis B virus; HCV, hepatitis C virus; HDL, high-density lipoprotein; IDU, injecting drug use; N(t)RTI, nucleoside/nucleotide reverse transcriptase inhibitor; PI, protease inhibitor.

Of the 1,415 people with a CKD event and 918 people with a CVD event, 154 (10.9%) experienced both types of event. Eighty-six (56%) of those who had both events experienced the CVD event first, and 67 (44%) the CKD event first. In 1 person the CKD and CVD event occurred on the same day. The majority of the CKD and CVD events (117, 76%) occurred 1 or more years apart.

### Overall CVD and CKD risk

The numbers of participants in each predicted risk group combination are expressed in Table 2. Of the 3,560 people with a 5-year predicted CVD risk > 5%, 3,331 (94%) were men and 229 (6%) were women. In these 3,331 men there were 528 CKD events (rate 23.0 per 1,000 pyrs), and in the 229 women there were 53 CKD events (rate 37.3 per 1,000 pyrs).

**Table 2:** Numbers of participants in each predicted risk group combination.

5-year CKD predicted risk	5-year CVD predicted risk		
	≤1%	>1%–5%	>5%
≤**1%**	6,225 (22.9%)	5,926 (21.9%)	383 (1.4%)
**>1%–5%**	2,047 (7.5%)	6,026 (22.1%)	1,592 (5.9%)
**>5%**	546 (2.0%)	2,865 (10.5%)	1,585 (5.8%)

CKD, chronic kidney disease; CVD, cardiovascular disease.

### CKD event rates according to CKD and CVD risk

**Fig 1:**
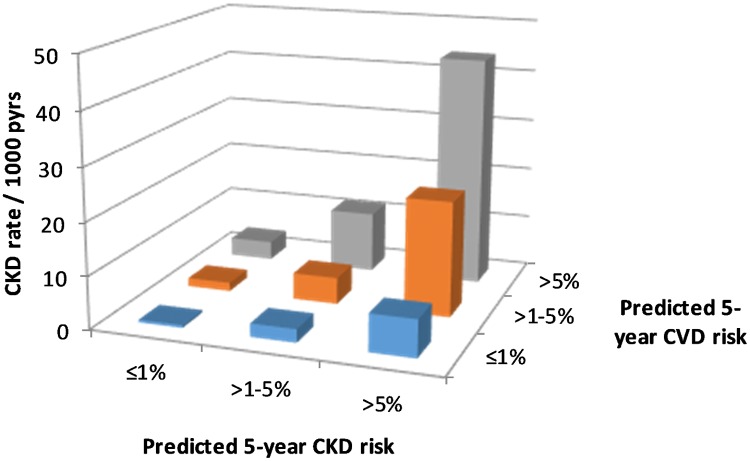
CKD event rates according to CKD and CVD risk. CKD, chronic kidney disease; CVD, cardiovascular disease; pyrs, person-years.

We observed 1,415 CKD events during follow-up, an overall rate of 7.00 per 1,000 pyrs (95% CI 6.6–7.7 per 1,000 pyrs). CKD event rates by predicted CKD and CVD risk group are shown in Fig 1, indicating that within each CKD risk group, the CKD event rate increases with higher predicted CVD risk.

This multiplicative effect of CKD and CVD predicted risk group on CKD events is confirmed by the Poisson regression models (Table 3), which show a highly statistically significant association between predicted CVD risk group and CKD events, after adjustment for CKD risk group. In these models there was no statistical evidence of an interaction between the predicted CKD and CVD risk groups, suggesting that the effects are multiplicative (i.e., additive on a log scale). This means, for example, that for an individual with a high predicted 5-year risk of both CKD and CVD, the incidence rate ratio (IRR) for CKD events would be 13.81 multiplied by 5.63, which equals 77.75, compared with an individual at low predicted risk for both CKD and CVD.

**Table 3:** Combined effect of CKD and CVD risk groups in predicting CKD and CVD events.

CKD and CVD risk group	IRR (95% CI)	*p*-Value	Interaction
***Predicting CKD events***			
CKD ≤1%	1.00		
CKD >1%–5%	3.46 (2.79, 4.30)	<0.001	
CKD >5%	13.81 (11.22, 17.01)	<0.001	
CVD ≤1%	1.00		
CVD >1%–5%	2.70 (2.16, 3.38)	<0.001	
CVD >5%	5.63 (4.47, 7.09)	<0.001	0.291
***Predicting CVD events***			
CVD ≤1%	1.00		
CVD >1%–5%	8.43 (5.91, 12.03)	<0.001	
CVD >5%	26.97 (18.68, 38.95)	<0.001	
CKD ≤1%	1.00		
CKD >1%–5%	1.19 (1.01, 1.44)	0.041	
CKD >5%	1.31 (1.09, 1.56)	0.005	0.329

Test for interaction is likelihood ratio test global *p*-value for non-additive effect (4 degrees of freedom).

CKD, chronic kidney disease; CVD, cardiovascular disease; IRR, incidence rate ratio.

The CKD event rates by predicted Framingham CVD and CKD risk strata were similar to the findings in the primary analysis (S1 Table).

### CVD event rates according to CKD and CVD risk groups

**Fig 2:**
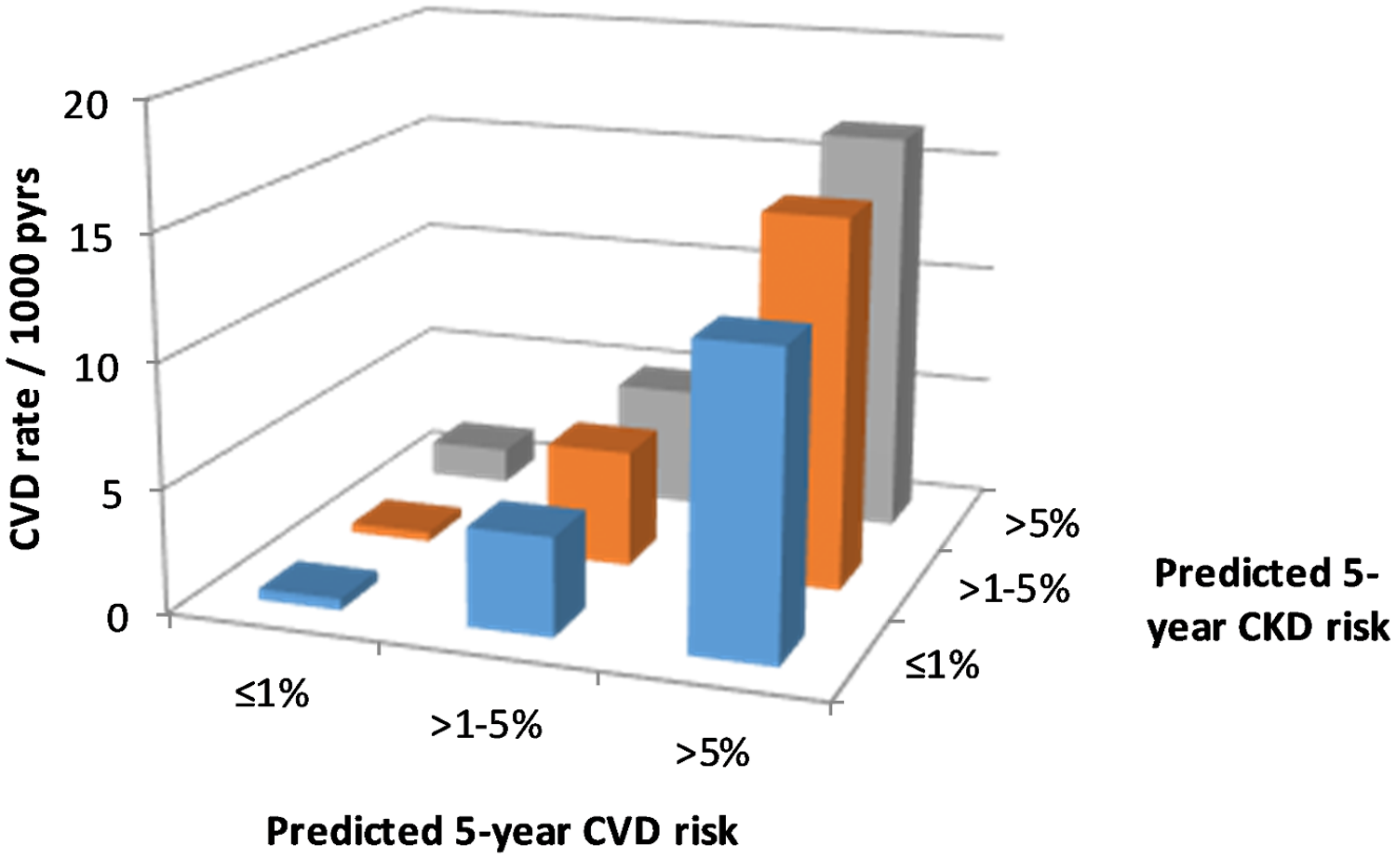
CVD event rates according to CKD and CVD risk. CKD, chronic kidney disease; CVD, cardiovascular disease; pyrs, person-years.

There were 918 CVD events documented during follow-up, an overall rate of 4.5 per 1,000 pyrs (95% CI 4.2–4.8 per 1,000 pyrs). CVD event rates by predicted CVD and CKD risk groups are shown in Fig 2. This figure shows large increases in CVD event rates with increasing predicted CVD risk, as would be expected. But there is also some evidence of increasing CVD events with increasing predicted CKD risk.

The Poisson regression models did confirm a statistically significant association between higher predicted CKD risk group and CVD events after adjustment for predicted CVD risk (Table 3). But the magnitude of this association was smaller than was seen for the association between predicted CVD risk group and CKD events. Again, there was no statistical evidence of an interaction between predicted CVD and CKD risk groups.

The CVD event rates by predicted Framingham CVD and CKD risk strata were also similar to the findings made in the primary analysis (S2 Table).

### Investigating covariates of the CVD risk score as predictors of CKD events

The relationship between the covariates of the CVD risk score and prediction of CKD events (adjusted for CKD risk group) is summarised in Table 4. We found that total plasma cholesterol level was associated with risk of a CKD event (IRR 1.48 per unit log total cholesterol, 95% CI 1.20, 1.83, *p <* 0.001), as were baseline CD4 count (IRR 0.90, 95% CI 0.86, 0.95, *p* < 0.001) and cumulative exposure to a PI (IRR 1.11 per year exposure, 95% CI 1.06, 1.15, *p <* 0.001) or to an N(t)RTI (IRR 1.05 per year exposure, 95% CI 1.03, 1.08, *p <* 0.001). We did not find statistically significant relationships for having a family history of CVD, being an ex- or current smoker, HDL cholesterol, or currently receiving abacavir.

**Table 4:** Covariates of CVD risk score as predictors for CKD events adjusted for CKD risk group.

Risk group or covariate	Adjusted only for CKD risk group		Fully adjusted model	
	IRR (95% CI)	*p*-Value	IRR (95% CI)	*p*-Value
CKD ≤1%	1.00		1.0	
CKD >1%–5%	4.92 (3.98, 6.09)	<0.001	4.49 (3.63, 5.57)	<0.001
CKD >5%	23.56 (19.30, 28.77)	<0.001	20.53 (16.77, 25.14)	<0.001
Family history of CVD (yes)	0.95 (0.80, 1.14)	0.617		
Current smoker	0.91 (0.80, 1.03)	0.121		
Ex-smoker	1.01 (0.88, 1.17)	0.861		
Ln total cholesterol	1.48 (1.20, 1.83)	<0.001	1.49 (1.20, 1.84)	<0.001
Ln HDL cholesterol	0.89 (0.77, 1.03)	0.121		
Ln base 2 CD4 count	0.90 (0.86, 0.95)	<0.001	0.87 (0.83, 0.91)	<0.001
Receiving abacavir	0.93 (0.81, 1.06)	0.287		
Cumulative PI use	1.11 (1.06, 1.15)	<0.001	1.04 (0.99, 1.10)	0.149
Cumulative N(t)RTI use	1.05 (1.03, 1.08)	<0.001	1.04 (1.02, 1.07)	0.002

CKD, chronic kidney disease; CVD, cardiovascular disease; HDL, high-density lipoprotein; IRR, incidence rate ratio; N(t)RTI, nucleoside/nucleotide reverse transcriptase inhibitor; PI, protease inhibitor.

In a multivariate analysis, after adjusting the covariates for each other as predictors of CKD events, we found similar associations as for the analysis adjusted only for CKD risk group, although the association for receiving a PI lost statistical significance (IRR 1.04, 95% CI 0.99, 1.10, *p* = 0.149).

### Investigating covariates of the CKD risk score as predictors of CVD events

After adjustment for CVD risk group, we found 1 covariate in the CKD risk score that was associated with CVD events (Table 5). Higher nadir CD4 count was associated with a reduced risk of CVD events: IRR 0.94 per 100 cells (95% CI 0.091, 0.98, *p* = 0.002). We did not find associations between predicted CKD risk and HIV exposure through injecting drug use, baseline eGFR, or being coinfected with viral hepatitis C.

**Table 5:** Covariates of CKD risk score as predictors for CVD events adjusted for CVD risk group.

Risk group or covariate	Adjusted only for CVD risk group	
	IRR (95% CI)	*p*-Value
CVD ≤1%	1.00	
CVD >1%–5%	9.00 (6.33, 12.80)	<0.001
CVD >5%	30.89 (21.65, 44.08)	<0.001
HIV exposure through IDU	1.00 (0.82, 1.21)	0.979
HCV positive	1.04 (0.88, 1.22)	0.653
eGFR (per 10 units)	1.00 (0.98, 1.03)	0.772
Nadir CD4 count (per 100 cells)	0.94 (0.91, 0.98)	0.002

CKD, chronic kidney disease; CVD, cardiovascular disease; eGFR, estimated glomerular filtration rate; HCV, hepatitis C virus; IDU, injecting drug use; IRR, incidence rate ratio.

### Diabetes by predicted CKD and CVD risk

Diabetes is a key covariate in both the CKD and CVD risk scores. Therefore, we were interested in exploring the prevalence of diabetes in the CKD and CVD predicted risk groups (Table 6). The prevalence of diabetes at baseline increased markedly with higher predicted CVD risk group. The association was less clear with CKD risk group, but of the 1,585 people at >5% predicted risk for both CVD and CKD, 366 (23.1%) had been diagnosed with diabetes.

**Table 6:** Diabetes prevalence at baseline by predicted CKD and CVD risk group.

5-year CVD risk group	5-year CKD risk group	*N*	*N* with diabetes (%)
≤1%	≤1%	6,225	23 (0.4%)
≤1%	>1%–5%	2,047	10 (0.5%)
≤1%	>5%	546	2 (0.4%)
>1%–5%	≤1%	5,946	61 (1.0%)
>1%–5%	>1%–5%	6,026	192 (3.2%)
>1%–5%	>5%	2,865	104 (3.6%)
>5%	≤1%	383	39 (10.2%)
>5%	>1%–5%	1,592	234 (14.7%)
>5%	>5%	1,585	366 (23.1%)
Overall		27,215	1,031 (3.8%)

CKD, chronic kidney disease; CVD, cardiovascular disease.

### Sensitivity analyses

We conducted sensitivity analyses assessing the predicted CKD and CVD risk scores as continuous measures rather than in groups. After adjustment for 5-year predicted CKD risk, 5-year CVD predicted risk was significantly associated with CKD events: IRR 1.065 per 1% increase (95% CI 1.058, 1.072, *p <* 0.001). Five-year CKD predicted risk was not significantly associated with CVD events after adjustment for 5-year CVD predicted risk: IRR 1.005 per 1% increase (95% CI 1.000, 1.011, *p* = 0.052). These analyses suggest that the multiplicative effects of predicted CKD and CVD risk groups on CKD and CVD events are not simply due to a loss of precision by categorising the predicted risks as ≤1%, >1%–5%, and >5%.

## Discussion

In this analysis we found that HIV-positive people with high predicted CVD or CKD risk were at significant risk for a future CVD or CKD event, and that this risk was multiplicative for those with greater degrees of risk. For instance, we observed a far higher CKD event rate for those at high risk (>5%) for both CVD and CKD (44.0 per 1,000 pyrs) compared to those at low risk for both (0.5 per 1,000 pyrs) and those at intermediate risk for both (5.1 per 1,000 pyrs). We found that the CKD event rate in those with a high CKD risk was highly sensitive to the degree of CVD risk, with a CKD event rate of 7.1, 21.9, and 44.0 events per 1,000 pyrs for a CVD risk of ≤1%, >1%–5%, and >5%, respectively. For CVD events we found that event rates were multiplicative in that there was a gradient from those at low risk for both events (0.45 per 1,000 pyrs) to those at high risk for both events (16.50 per 1,000 pyrs), with those at intermediate risk in between.

It is widely acknowledged that there are complex relationships between CVD and CKD, and that the risks that mediate both of these conditions can interact, leading to more severe illness and poorer prognosis [10]. Our results are consistent with this understanding in that we found that both chronic diseases were associated with an elevated risk for the other. Overall our results suggest that among people living with HIV, the association between CVD risk and a future CKD event is stronger than the association between CKD risk and a future CVD event, and that the strength of the association may be particularly marked in those who are at high risk for both CVD and CKD. This is a novel finding, as the opposite has consistently been noted in the general population, where those with CKD are at markedly elevated risk for CVD. For the general population, for instance, when adjusted for traditional cardiovascular risk factors, impaired kidney function and raised concentrations of albumin in urine increase the risk of CVD 2- to 4-fold [11]. In interpreting these findings, it should be noted that this difference may be a function of the difference in the definition of the 2 disease endpoints used in the study; CVD is a clinical event adjudicated centrally according to specific criteria, whereas CKD is defined by the presence of impaired renal function as indicated by a laboratory biomarker (eGFR) observed over 2 consecutive occasions at least 3 months apart. This laboratory-based definition is thereby a sensitive measurement that captures people with true CKD but may also include people with transient decreases in eGFR not indicative of irreversible renal function deterioration.

In terms of the risk factors for development of CKD events in those with CVD risk, we found that total cholesterol level and cumulative PI and N(t)RTI use raised the risk of CKD events, and that a higher baseline CD4 count was associated with a lower rate of future CKD events. These findings are consistent with results previously reported in the D:A:D cohort [15–18]. We found that cumulative exposure to N(t)RTIs predicts CKD events, despite tenofovir exposure being included as an adjustment in the CKD predicted risk score. This may be because tenofovir exposure was included in the CKD predicted risk model as an acute effect rather than an effect that increases with increasing exposure.

For CVD events according to CKD risk, we did not find an association between eGFR and CVD risk. This is not consistent with findings in the general community. However, the failure to find such an association may be due to effective management of renal insufficiency, with a resultant decrease in the risk of CVD in this population. This finding also seems inconsistent with previous D:A:D analyses [12]. This apparent inconsistency is likely explained by the fact that the present analysis was based on a single baseline eGFR with participants selected with eGFR in a relatively healthy range (>60 ml/min/1.73 m^2^), whereas previous analyses were time updated and included far lower eGFR values.

It is recognised that both CKD and CVD are largely preventable conditions and that modifying and controlling their risk factors will reduce the risk of onset of both diseases. Despite this evidence and much effort over the past decade to focus attention on reducing CVD risk in HIV-positive people, a lack of attention to the management of CVD in people living with HIV has been documented [19]. A recent modelling analysis within the ATHENA cohort examined the impact of several interventions, such as earlier antiretroviral treatment, smoking cessation, and anti-hypertensive or lipid-lowering treatment, on future CVD events among HIV-positive people [20]. The results suggested that all the selected interventions, but especially smoking cessation and blood pressure and serum lipid control, would have a positive effect in reducing CVD.

We performed an analysis that assessed the extent to which the presence of diabetes in the participants may provide at least a partial explanation of the multiplicative risks we observed in those with higher degrees of risk, and particularly in those with a high risk for both events. We found that nearly a quarter of those with a high 5-year CKD and CVD risk had been diagnosed with diabetes, compared to very few individuals diagnosed with diabetes (0.4%) in the low CKD and CVD risk groups. This confirms that diabetes is a powerful risk factor for poor outcomes in HIV-positive people, as in the general community, and it behoves all clinicians to adequately screen, diagnose, treat, and, above all, prevent this complication to facilitate the optimal longevity and quality of life in HIV-positive people.

Our study has some limitations. Prediction models are limited by restrictions in the available data, and some variables that may affect CKD and CVD risk were not available for analysis (e.g., inflammatory markers). In this analysis, the risk equations were applied to the datasets that were largely used to develop them. It may be, therefore, that the equations predict more accurately in D:A:D data than they would in other independent datasets, and this may limit the generalisability of our findings. Both equations, however, were validated: the CVD equation using internal—external cross-validation, and the CKD equation on independent datasets. The CVD and CKD endpoints used in this analysis are quite different. CVD is a serious clinical event, subject to central validation, whereas CKD is an earlier, less serious event based on a confirmed laboratory marker. As noted above, compared with the more specific adjudicated CVD endpoint, the CKD definition may be capturing a broader set of events that might include true episodes of chronic and progressive CKD but also transient events in people who may be temporarily systemically unwell, hospitalised, and/or undergoing a medical procedure, all of which may lead to episodic decreases in eGFR without this necessarily resulting in persistent renal dysfunction. We attempted to minimise the possibility of miscategorising people with CKD by requiring there to be at least 2 consecutive eGFR measurements ≤ 60 ml/min/1.73 m^2^ to make the diagnosis. However, the ‘softer’ definition of CKD may at least partially explain the finding that the magnitude of association was greater when analysing CKD events by CVD risk than when analysing CVD events by CKD risk. It is uncertain how the results might have differed if a hard clinical endpoint for renal disease, such as ESRD, had been used. Such an approach, however, was not possible in our analyses, as a prediction tool for ESRD in HIV-positive people is not available, and the number of ESRD events in D:A:D is not large. In the D:A:D database, we are unable to distinguish cases of either CKD or CVD that were a direct result of a previous event.

In conclusion, we have shown that HIV-positive people not uncommonly have high predicted CVD and/or CKD risk, and that these interact to create substantial risks for future morbid events. We found that people at high predicted risk for both CVD and CKD have substantially greater risks for both CVD and CKD compared with those at low predicted risk for both, and those at high predicted risk for only CVD or only CKD. This suggests that CVD and CKD risk in HIV-positive persons should be assessed together. These data also suggest that the primary prevention and effective management of these comorbidities, prioritising those interventions that have been repeatedly shown to be effective in the general population, will convey the same if not greater benefits for the population of HIV-positive people. Primary prevention and the effective management of comorbidities should be incorporated into the development of guidelines and defining future research priorities for HIV-positive people.

## Supporting information

S1 STROBE Checklist (DOC)

S1 Table CKD event rates by predicted Framingham CVD and CKD risk strata. (DOCX)

S2 Table CVD event rates according to predicted Framingham CVD and CKD risk strata. (DOCX)

S1 Text Full list of members of the D:A:D Steering Committee and Study Group. (DOCX)

S2 Text D:A:D concept study sheet. (DOC)

## Abbreviations

BP: blood pressure
CKD: chronic kidney disease
CVD: cardiovascular disease
D:A:D: Data Collection on Adverse Events of Anti-HIV Drugs
eGFR: estimated glomerular filtration rate
ESRD: end-stage renal disease
HBV: hepatitis B virus
HCV: hepatitis C virus
HDL: high-density lipoprotein
IDU: injecting drug use
IRR: incidence rate ratio
N(t)RTI: nucleoside/nucleotide reverse transcriptase inhibitor
PI: protease inhibitor
pyrs: person-years
