# Supplementary material for: Cardiovascular disease (CVD) and chronic kidney disease (CKD) event rates in HIV-positive persons at high predicted CVD and CKD risk: A prospective analysis of the D:A:D observational study
Source: PLoS Med. 2017 Nov 7;14(11):e1002424. doi: 10.1371/journal.pmed.1002424 (PMC5675358; doi:10.1371/journal.pmed.1002424)
Supplement: S1 Table — (DOCX) [file pmed.1002424.s002.docx]

| **S1 Table. CKD event rates by predicted Framingham CVD and CKD risk strata.** | | | | | |
| --- | --- | --- | --- | --- | --- |
| **5-year CVD risk strata** | **5-year CKD risk strata** | **N (%)** | **Events** | **Pyrs** | **Rate per 1000 pyrs** |
| ≤1% | ≤1% | 6343 (23.1%) | 26 | 47690 | 0.55 |
| ≤1% | 1-5% | 2033 (7.5%) | 36 | 15400 | 2.34 |
| ≤1% | >5% | 499 (1.8%) | 34 | 3685 | 9.22 |
| 1-5% | ≤1% | 5984 (22.0%) | 73 | 46388 | 1.57 |
| 1-5% | 1-5% | 6411 (23.6%) | 255 | 48096 | 5.30 |
| 1-5% | >5% | 3157 (11.6%) | 475 | 22192 | 21.40 |
| >5% | ≤1% | 227 (0.8%) | 9 | 1748 | 5.15 |
| >5% | 1-5% | 1221 (4.5%) | 109 | 8591 | 12.69 |
| >5% | >5% | 1340 (4.9%) | 398 | 8275 | 48.1 |
| Overall | | 27215 | 1415 | 202064 | 7.00 |
