# Supplementary material for: Cardiovascular disease (CVD) and chronic kidney disease (CKD) event rates in HIV-positive persons at high predicted CVD and CKD risk: A prospective analysis of the D:A:D observational study
Source: PLoS Med. 2017 Nov 7;14(11):e1002424. doi: 10.1371/journal.pmed.1002424 (PMC5675358; doi:10.1371/journal.pmed.1002424)
Supplement: S2 Table — (DOCX) [file pmed.1002424.s003.docx]

| **S2 Table. CVD event rates according to predicted Framingham CVD and CKD risk strata.** | | | | | |
| --- | --- | --- | --- | --- | --- |
| **5-year CVD risk strata** | **5-year CKD risk strata** | **N (%)** | **Events** | **Pyrs** | **Rate per 1000 pyrs** |
| ≤1% | ≤1% | 6343 (23.1%) | 28 | 47657 | 0.59 |
| ≤1% | 1-5% | 2033 (7.5%) | 17 | 15464 | 1.10 |
| ≤1% | >5% | 499 (1.8%) | 8 | 3788 | 2.11 |
| 1-5% | ≤1% | 5984 (22.0%) | 190 | 45868 | 4.14 |
| 1-5% | 1-5% | 6411 (23.6%) | 247 | 47955 | 5.15 |
| 1-5% | >5% | 3157 (11.6%) | 135 | 23643 | 5.71 |
| >5% | ≤1% | 227 (0.8%) | 20 | 1703 | 11.74 |
| >5% | 1-5% | 1221 (4.5%) | 122 | 8467 | 14.41 |
| >5% | >5% | 1340 (4.9%) | 151 | 9313 | 16.21 |
| Overall | | 27215 | 918 | 203859 | 4.50 |
