# Supplementary material for: Cardiovascular disease (CVD) and chronic kidney disease (CKD) event rates in HIV-positive persons at high predicted CVD and CKD risk: A prospective analysis of the D:A:D observational study
Source: PLoS Med. 2017 Nov 7;14(11):e1002424. doi: 10.1371/journal.pmed.1002424 (PMC5675358; doi:10.1371/journal.pmed.1002424)
Supplement: S1 Text — (DOCX) [file pmed.1002424.s004.docx]

**Full Acknowledgement List**

D:A:D Participating Cohorts

Aquitaine, France; CPCRA, USA; NICE Cohort, France; ATHENA, The Netherlands; EuroSIDA, Europe; SHCS, Switzerland, AHOD, Australia; HIV-BIVUS, Sweden; St.Pierre Brussels Cohort, Belgium; BASS, Spain, The ICONA Foundation, Italy

D:A:D Steering Committee: Names marked with *, Chair with ¢

Cohort PIs: W El-Sadr* (CPCRA), G Calvo* (BASS), F Bonnet and F Dabis* (Aquitaine), O Kirk* and A Mocroft* (EuroSIDA), M Law* (AHOD), A d’Arminio Monforte* (ICONA), L Morfeldt* (HivBIVUS), C Pradier* (Nice), P Reiss* (ATHENA), R Weber* (SHCS), S De Wit* (Brussels)

Cohort coordinators and data managers: A Lind-Thomsen (coordinator), R Salbøl Brandt, M Hillebreght, S Zaheri, FWNM Wit (ATHENA), A Scherrer, F Schöni-Affolter, M Rickenbach (SHCS), A Tavelli, I Fanti (ICONA), O Leleux, J Mourali, F Le Marec, E Boerg (Aquitaine), E Thulin, A Sundström (HIVBIVUS), G Bartsch, G Thompsen (CPCRA), C Necsoi, M Delforge (Brussels), E Fontas, C Caissotti, K Dollet (Nice), S Mateu, F Torres (BASS), K Petoumenos, A Blance, R Huang, R Puhr (AHOD), K Grønborg Laut, D Kristensen (EuroSIDA)

Statisticians: CA Sabin*, AN Phillips*, DA Kamara, CJ Smith, A Mocroft*

D:A:D coordinating office: CI Hatleberg, L Ryom, A Lind-Thomsen, RS Brandt, D Raben, C Matthews, A Bojesen, AL Grevsen, JD Lundgren*¢

Member of the D:A:D Oversight Committee: B Powderly*, N Shortman*, C Moecklinghoff*, G Reilly*, X Franquet*

D:A:D working group experts:

Kidney: L Ryom, A Mocroft*, O Kirk *, P Reiss*, C Smit, M Ross, CA Fux, P Morlat, E Fontas, DA Kamara, CJ Smith, JD Lundgren *¢

Mortality: CJ Smith, L Ryom, CI Hatleberg, AN Phillips*, R Weber*, P Morlat, C Pradier*, P Reiss*, FWNM Wit, N Friis-Møller, J Kowalska, JD Lundgren*¢

Cancer: CA Sabin*, L Ryom, CI Hatleberg, M Law*, A d'Arminio Monforte*, F Dabis*, F Bonnet*, P Reiss*, FWNM Wit, CJ Smith, DA Kamara, J Bohlius, M Bower, G Fätkenheuer, A Grulich, JD Lundgren*¢

External endpoint reviewers: A Sjøl (CVD), P Meidahl (oncology), JS Iversen (nephrology)

Funding: ‘Oversight Committee for The Evaluation of Metabolic Complications of HAART’ with representatives from academia, patient community, FDA, EMA and a consortium of AbbVie, Bristol-Myers Squibb, Gilead Sciences, ViiV Healthcare, Merck and Janssen Pharmaceuticals.

The current members of the 11 Cohorts are as follows:

ATHENA (AIDS Therapy Evaluation Project Netherlands):

Central coordination: P. Reiss*, S. Zaheri, M Hillebregt, F.W.N.M. Wit;

CLINICAL CENTRES (¤ denotes site coordinating physician) Academic Medical Centre of the University of Amsterdam: J.M. Prins¤, T.W. Kuijpers, H.J. Scherpbier, J.T.M. van der Meer, F.W.N.M. Wit, M.H. Godfried, P. Reiss, T. van der Poll, F.J.B. Nellen, S.E. Geerlings, M. van Vugt, D. Pajkrt, J.C. Bos, W.J. Wiersinga, M. van der Valk, A. Goorhuis, J.W. Hovius, J. van Eden, A. Henderiks, A.M.H. van Hes, M. Mutschelknauss, H.E. Nobel, F.J.J. Pijnappel, S. Jurriaans, N.K.T. Back, H.L. Zaaijer, B. Berkhout, M.T.E. Cornelissen, C.J. Schinkel, X.V. Thomas. Admiraal De Ruyter Ziekenhuis, Goes: M. van den Berge, A. Stegeman, S. Baas, L. Hage de Looff, D. Versteeg. Catharina Ziekenhuis, Eindhoven: M.J.H. Pronk¤, H.S.M. Ammerlaan, E.S. de Munnik. A.R. Jansz, J. Tjhie, M.C.A. Wegdam, B. Deiman, V. Scharnhorst. Emma Kinderziekenhuis: A. van der Plas, A.M. Weijsenfeld. Erasmus MC, Rotterdam: M.E. van der Ende¤, T.E.M.S. de Vries-Sluijs, E.C.M. van Gorp, C.A.M. Schurink, J.L. Nouwen, A. Verbon, B.J.A. Rijnders, H.I. Bax, M. van der Feltz. N. Bassant, J.E.A. van Beek, M. Vriesde, L.M. van Zonneveld. A. de Oude-Lubbers, H.J. van den Berg-Cameron, F.B. Bruinsma-Broekman, J. de Groot, M. de Zeeuw- de Man, C.A.B. Boucher, M.P.G Koopmans, J.J.A van Kampen, S.D. Pas. Erasmus MC–Sophia, Rotterdam: G.J.A. Driessen, A.M.C. van Rossum, L.C. van der Knaap, E. Visser. Flevoziekenhuis, Almere: J. Branger¤, A. Rijkeboer-Mes, C.J.H.M. Duijf-van de Ven. HagaZiekenhuis, Den Haag: E.F. Schippers¤, C. van Nieuwkoop. J.M. van IJperen, J. Geilings. G. van der Hut. P.F.H. Franck. HIV Focus Centrum (DC Klinieken): A. van Eeden¤. W. Brokking, M. Groot, L.J.M. Elsenburg, M. Damen, I.S. Kwa. Isala, Zwolle: P.H.P. Groeneveld¤, J.W. Bouwhuis, J.F. van den Berg, A.G.W. van Hulzen, G.L. van der Bliek, P.C.J. Bor, P. Bloembergen, M.J.H.M. Wolfhagen, G.J.H.M. Ruijs. Leids Universitair Medisch Centrum, Leiden: , F.P. Kroon¤, M.G.J. de Boer, M.P. Bauer, H. Jolink, A.M. Vollaard, W. Dorama, N. van Holten, E.C.J. Claas, E. Wessels. Maasstad Ziekenhuis, Rotterdam: J.G. den Hollander¤, K. Pogany, A. Roukens, M. Kastelijns, J.V. Smit, E. Smit, D. Struik-Kalkman, C. Tearno, M. Bezemer, T. van Niekerk, O. Pontesilli. Maastricht UMC+, Maastricht: S.H. Lowe¤, A.M.L. Oude Lashof, D. Posthouwer, R.P. Ackens, J. Schippers, R. Vergoossen, B. Weijenberg-Maes, I.H.M. van Loo, T.R.A. Havenith. MCH-Bronovo, Den Haag: E.M.S. Leyten¤, L.B.S. Gelinck, A. van Hartingsveld, C. Meerkerk, G.S. Wildenbeest, J.A.E.M. Mutsaers, C.L. Jansen. MC Slotervaart, Amsterdam: J.W. Mulder, S.M.E. Vrouenraets, F.N. Lauw, M.C. van Broekhuizen, H. Paap, D.J. Vlasblom, P.H.M. Smits. MC Zuiderzee, Lelystad: S. Weijer¤, R. El Moussaoui, A.S. Bosma. Medisch Centrum Leeuwarden, Leeuwarden: M.G.A.van Vonderen¤, D.P.F. van Houte, L.M. Kampschreur, K. Dijkstra, S. Faber, J Weel. Medisch Spectrum Twente, Enschede: G.J. Kootstra¤, C.E. Delsing, M. van der Burg-van de Plas, H. Heins, E. Lucas. Noorwest Ziekenhuisgroep, Alkmaar: W. Kortmann¤, G. van Twillert¤, J.W.T. Cohen Stuart, B.M.W. Diederen, D. Pronk, F.A. van Truijen-Oud, W. A. van der Reijden, R. Jansen. OLVG, Amsterdam: K. Brinkman¤, G.E.L. van den Berk, W.L. Blok, P.H.J. Frissen, K.D. Lettinga W.E.M. Schouten, J. Veenstra, C.J. Brouwer, G.F. Geerders, K. Hoeksema, M.J. Kleene, I.B. van der Meché, M. Spelbrink, H. Sulman, A.J.M. Toonen, S. Wijnands, M. Damen, D. Kwa, E. Witte. Radboudumc, Nijmegen: P.P. Koopmans, M. Keuter, A.J.A.M. van der Ven, H.J.M. ter Hofstede, A.S.M. Dofferhoff, R. van Crevel, M. Albers, M.E.W. Bosch, K.J.T. Grintjes-Huisman, B.J. Zomer, F.F. Stelma, J. Rahamat-Langendoen,D. Burger. Rijnstate, Arnhem: C. Richter¤, E.H. Gisolf, R.J. Hassing, G. ter Beest, P.H.M. van Bentum, N. Langebeek, R. Tiemessen, C.M.A. Swanink. Spaarne Gasthuis, Haarlem: S.F.L. van Lelyveld¤, R. Soetekouw, N. Hulshoff, L.M.M. van der Prijt, J. van der Swaluw, N. Bermon, W.A. van der Reijden, R. Jansen, B.L. Herpers, D.Veenendaal. Medisch Centrum Jan van Goyen, Amsterdam: D.W.M. Verhagen, M. van Wijk. St Elisabeth Ziekenhuis, Tilburg: M.E.E. van Kasteren¤, A.E. Brouwer, B.A.F.M. de Kruijf-van de Wiel, M. Kuipers, R.M.W.J. Santegoets, B. van der Ven, J.H. Marcelis, A.G.M. Buiting, P.J. Kabel. Universitair Medisch Centrum Groningen, Groningen: W.F.W. Bierman¤, H. Scholvinck, K.R. Wilting, Y. Stienstra, H. de Groot-de Jonge, P.A. van der Meulen, D.A. de Weerd, J. Ludwig-Roukema, H.G.M. Niesters, A. Riezebos-Brilman, C.C. van Leer-Buter, M. Knoester. Universitair Medisch Centrum Utrecht, Utrecht: A.I.M. Hoepelman¤, T. Mudrikova, P.M. Ellerbroek, J.J. Oosterheert, J.E. Arends, R.E. Barth, M.W.M. Wassenberg, E.M. Schadd, D.H.M. van Elst-Laurijssen, E.E.B. van Oers-Hazelzet, S. Vervoort, M. van Berkel, R. Schuurman, F. Verduyn-Lunel, A.M.J. Wensing. VUmc, Amsterdam: E.J.G. Peters¤, M.A. van Agtmael, M. Bomers, J. de Vocht, M. Heitmuller, L.M. Laan, A.M. Pettersson, C.M.J.E. Vandenbroucke-Grauls, C.W. Ang. Wilhelmina Kinderziekenhuis, UMCU, Utrecht: S.P.M. Geelen, T.F.W. Wolfs, L.J. Bont, N. Nauta. COORDINATING CENTRE P. Reiss, D.O. Bezemer, A.I. van Sighem, C. Smit, F.W.N.M. Wit., T.S. Boender, S. Zaheri, M. Hillebregt, A. de Jong, D. Bergsma, P. Hoekstra, A. de Lang, S. Grivell, A. Jansen, M.J. Rademaker, M. Raethke, R. Meijering, S. Schnörr, L. de Groot, M. van den Akker, Y. Bakker, E. Claessen, A. El Berkaoui, J. Koops, E. Kruijne, C. Lodewijk, L. Munjishvili, B. Peeck, C. Ree, R. Regtop, Y. Ruijs, T. Rutkens, L. van de Sande, M. Schoorl, A. Timmerman, E. Tuijn, L. Veenenberg, S. van der Vliet, A. Wisse, T. Woudstra, B. Tuk.

Aquitaine Cohort (France)

Composition du Conseil scientifique :

Coordination: F. Bonnet, F. Dabis

Scientific committee: M. Dupon, V. Gaborieau, D. Lacoste, D. Malvy, P. Mercié, P. Morlat, D. Neau, JL. Pellegrin, S. Tchamgoué, E. Lazaro, C. Cazanave, M. Vandenhende, M.O. Vareil, Y. Gérard, P. Blanco, S. Bouchet, D. Breilh, H. Fleury, I. Pellegrin, G. Chêne, R. Thiébaut, L. Wittkop, L. Wittkop, O. Leleux, S. Lawson-Ayayi, A. Gimbert, S. Desjardin, L. Lacaze-Buzy, V. Petrov-Sanchez

Epidemiology and Methodology: F. Bonnet, G. Chêne, F. Dabis, R. Thiébaut, L. Wittkop

Infectious Diseases and Internal Medicine: K. André, N. Bernard, F. Bonnet, O. Caubet, L. Caunegre, C. Cazanave, I. Chossat, C. Courtault, FA. Dauchy, S. De Witte, D. Dondia, M. Dupon, P. Duffau, H. Dutronc, S. Farbos, I. Faure, H. Ferrand, V. Gaborieau, Y. Gerard, C. Greib, M. Hessamfar, Y. Imbert, D. Lacoste , P. Lataste, E. Lazaro, D. Malvy, J. Marie, M. Mechain, P. Mercié, E.Monlun, P. Morlat, D. Neau, A. Ochoa, JL. Pellegrin, T. Pistone, I. Raymond,MC. Receveur, P. Rispal, L. Sorin, S. Tchamgoué, C. Valette, MA. Vandenhende, MO. Vareil, JF. Viallard, H. Wille, G. Wirth.

Immunology: I. Pellegrin, P. Blanco

Virology: H. Fleury, Me. Lafon, P. Trimoulet, P. Bellecave, C. Tumiotto

Pharmacology: S. Bouchet, D. Breilh, F. Haramburu, G. Miremeont-Salamé

Data collection, Project Management and Statistical Analyses: MJ. Blaizeau, M. Decoin, C. Hannapier, E. Lenaud et A. Pougetoux; S. Delveaux, C. D’Ivernois, F. Diarra B. Uwamaliya-Nziyumvira, O. Leleux; F. Le Marec, Eloïse Boerg, S. Lawson-Ayayi;

IT department and eCRF development: G. Palmer, V. Conte, V. Sapparrart

AHOD (Australian HIV Observational Database, Australia):

Central coordination: M. Law *, K. Petoumenos, R Puhr, R Huang (Sydney, New South Wales). Participating physicians (city, state): R. Moore, S. Edwards, J. Hoy, K. Watson, N. Roth, H Lau (Melbourne, Victoria); M Bloch, D. Baker, A. Carr, D. Cooper, (Sydney, New South Wales);M O’Sullivan (Gold Coast, Queensland), D. Nolan, G Guelfi (Perth, Western Australia).

BASS (Spain):

Central coordination: G. Calvo, F. Torres, S. Mateu (Barcelona);

Participating physicians (city): P. Domingo, M.A. Sambeat, J. Gatell, E. Del Cacho, J. Cadafalch, M. Fuster (Barcelona); C. Codina, G. Sirera, A. Vaqué (Badalona).

The Brussels St Pierre Cohort (Belgium):

Coordination: S. De Wit*, N. Clumeck, M. Delforge, C. Necsoi.

Participating physicians: N. Clumeck, S. De Wit*, AF Gennotte, M. Gerard, K. Kabeya, D. Konopnicki, A. Libois, C. Martin, M.C. Payen, P. Semaille, Y. Van Laethem.

CPCRA (USA):

Central coordination: J. Neaton, G. Bartsch, W.M. El-Sadr*, E. Krum, G. Thompson, D. Wentworth;

Participating physicians (city, state): R. Luskin-Hawk (Chicago, Illinois); E. Telzak (Bronx, New York); W.M. El-Sadr (Harlem, New York); D.I. Abrams (San Francisco, California); D. Cohn (Denver, Colorado); N. Markowitz (Detroit, Michigan); R. Arduino (Houston, Texas); D. Mushatt (New Orleans, Louisiana); G. Friedland (New Haven, Connecticut); G. Perez (Newark, New Jersey); E. Tedaldi (Philadelphia, Pennsylvania); E. Fisher (Richmond, Virginia); F. Gordin (Washington, DC); L.R. Crane (Detroit, Michigan); J. Sampson (Portland, Oregon); J. Baxter (Camden, New Jersey).

EuroSIDA (multinational)

Steering Committee: J Gatell, B Gazzard, A Horban, I Karpov, M Losso, A d’Arminio Monforte, C Pedersen, M Ristola, A Phillips, P Reiss, J Lundgren, J Rockstroh

Chair: J Rockstroh

Study Co-leads: A Mocroft, O Kirk

Coordinating Centre Staff: O Kirk, L Peters, C Matthews, AH Fischer, A Bojesen, D Raben, D Kristensen, K Grønborg Laut, JF Larsen, D Podlekareva

Statistical Staff: A Mocroft, A Phillips, A Cozzi-Lepri, L Shepherd, A Schultze, S Amele

The multi-centre study group, EuroSIDA (national coordinators in parenthesis).

Argentina: (M Losso), M Kundro, Hospital JM Ramos Mejia, Buenos Aires.

Austria: (B Schmied), Pulmologisches Zentrum der Stadt Wien, Vienna; R Zangerle, Medical University Innsbruck, Innsbruck.

Belarus: (I Karpov), A Vassilenko, Belarus State Medical University, Minsk, VM Mitsura, Gomel State Medical University, Gomel; D Paduto, Regional AIDS Centre, Svetlogorsk.

Belgium: (N Clumeck), S De Wit, M Delforge, Saint-Pierre Hospital, Brussels; E Florence, Institute of Tropical Medicine, Antwerp; L Vandekerckhove, University Ziekenhuis Gent, Gent.

Bosnia-Herzegovina: (V Hadziosmanovic), Klinicki Centar Univerziteta Sarajevo, Sarajevo.

Croatia: (J Begovac), University Hospital of Infectious Diseases, Zagreb.

Czech Republic: (L Machala), D Jilich, Faculty Hospital Bulovka, Prague; D Sedlacek, Charles University Hospital, Plzen.

Denmark: G Kronborg,T Benfield, Hvidovre Hospital, Copenhagen; J Gerstoft, T Katzenstein, Rigshospitalet, Copenhagen; NF Møller, C Pedersen, Odense University Hospital, Odense; L Ostergaard, Skejby Hospital, Aarhus, L Wiese, Roskilde Hospital, Roskilde; L N Nielsen, Hillerod Hospital, Hillerod.

Estonia: (K Zilmer), West-Tallinn Central Hospital, Tallinn; Jelena Smidt, Nakkusosakond Siseklinik, Kohtla-Järve.

Finland: (M Ristola), I Aho, Helsinki University Central Hospital, Helsinki.

France: (J-P Viard), Hôtel-Dieu, Paris; P-M Girard, Hospital Saint-Antoine, Paris; C Pradier, E Fontas, Hôpital de l'Archet, Nice; C Duvivier, Hôpital Necker-Enfants Malades, Paris.

Germany: (J Rockstroh), Universitäts Klinik Bonn; R Schmidt, Medizinische Hochschule Hannover; O Degen, University Medical Center Hamburg-Eppendorf, Infectious Diseases Unit, Hamburg; HJ Stellbrink, IPM Study Center, Hamburg; C Stefan, JW Goethe University Hospital, Frankfurt; J Bogner, Medizinische Poliklinik, Munich; G. Fätkenheuer, Universität Köln, Cologne.

Georgia: (N Chkhartishvili) Infectious Diseases, AIDS & Clinical Immunology Research Center, Tbilisi

Greece: (P Gargalianos), G Xylomenos, K Armenis, Athens General Hospital "G Gennimatas"; H Sambatakou, Ippokration General Hospital, Athens.

Hungary: (J Szlávik), Szent Lásló Hospital, Budapest.

Iceland: (M Gottfredsson), Landspitali University Hospital, Reykjavik.

Ireland: (F Mulcahy), St. James's Hospital, Dublin.

Israel: (I Yust), D Turner, M Burke, Ichilov Hospital, Tel Aviv; E Shahar, G Hassoun, Rambam Medical Center, Haifa; H Elinav, M Haouzi, Hadassah University Hospital, Jerusalem; D Elbirt, ZM Sthoeger, AIDS Center (Neve Or), Jerusalem.

Italy: (A D’Arminio Monforte), Istituto Di Clinica Malattie Infettive e Tropicale, Milan; R Esposito, I Mazeu, C Mussini, Università Modena, Modena; F Mazzotta, A Gabbuti, Ospedale S Maria Annunziata, Firenze; V Vullo, M Lichtner, University di Roma la Sapienza, Rome; M Zaccarelli, A Antinori, R Acinapura, M Plazzi, Istituto Nazionale Malattie Infettive Lazzaro Spallanzani, Rome; A Lazzarin, A Castagna, N Gianotti, Ospedale San Raffaele, Milan; M Galli, A Ridolfo, Osp. L. Sacco, Milan.

Latvia: (B Rozentale), Infectology Centre of Latvia, Riga.

Lithuania: (V Uzdaviniene) Vilnius University Hospital Santariskiu Klinikos, Vilnius; R Matulionyte, Center of Infectious Diseases, Vilnius University Hospital Santariskiu Klinikos, Vilnius.

Luxembourg: (T Staub), R Hemmer, Centre Hospitalier, Luxembourg.

Netherlands: (P Reiss), Academisch Medisch Centrum bij de Universiteit van Amsterdam, Amsterdam.

Norway: (V Ormaasen), A Maeland, J Bruun, Ullevål Hospital, Oslo.

Poland: (B Knysz), J Gasiorowski, M Inglot, Medical University, Wroclaw; A Horban, E Bakowska, Centrum Diagnostyki i Terapii AIDS, Warsaw; R Flisiak, A Grzeszczuk, Medical University, Bialystok; M Parczewski, K Maciejewska, B Aksak-Was, Medical Univesity, Szczecin; M Beniowski, E Mularska, Osrodek Diagnostyki i Terapii AIDS, Chorzow; T Smiatacz, M Gensing, Medical University, Gdansk; E Jablonowska, E Malolepsza, K Wojcik, Wojewodzki Szpital Specjalistyczny, Lodz; I Mozer-Lisewska, Poznan University of Medical Sciences, Poznan.

Portugal: (L Caldeira), Hospital Santa Maria, Lisbon; K Mansinho, Hospital de Egas Moniz, Lisbon; F Maltez, Hospital Curry Cabral, Lisbon.

Romania: (R Radoi), C Oprea, Spitalul de Boli Infectioase si Tropicale: Dr. Victor Babes, Bucarest.

Russia: (A Panteleev), O Panteleev, St Petersburg AIDS Centre, St Peterburg; A Yakovlev, Medical Academy Botkin Hospital, St Petersburg; T Trofimora, Novgorod Centre for AIDS, Novgorod, I Khromova, Centre for HIV/AIDS & and Infectious Diseases, Kaliningrad; E Kuzovatova, Nizhny Novgorod Scientific and Research Institute of Epidemiology and Microbiology named after Academician I.N. Blokhina, Nizhny Novogrod; E Borodulina, E Vdoushkina, Samara State Medical University, Samara.

Serbia: (D Jevtovic), The Institute for Infectious and Tropical Diseases, Belgrade.

Slovenia: (J Tomazic), University Clinical Centre Ljubljana, Ljubljana.

Spain: (JM Gatell), JM Miró, Hospital Clinic Universitari de Barcelona, Barcelona; S Moreno, J. M. Rodriguez, Hospital Ramon y Cajal, Madrid; B Clotet, A Jou, R Paredes, C Tural, J Puig, I Bravo, Hospital Germans Trias i Pujol, Badalona; P Domingo, M Gutierrez, G Mateo, MA Sambeat, Hospital Sant Pau, Barcelona; JM Laporte, Hospital Universitario de Alava, Vitoria-Gasteiz.

Sweden: (K Falconer), A Thalme, A Sonnerborg, Karolinska University Hospital, Stockholm; A Blaxhult, Venhälsan-Sodersjukhuset, Stockholm; L Flamholc, Malmö University Hospital, Malmö.

Switzerland: (A Scherrer), R Weber, University Hospital Zurich; M Cavassini, University Hospital Lausanne; A Calmy, University Hospital Geneva; H Furrer, University Hospital Bern; M Battegay, University Hospital Basel; P Schmid, Cantonal Hospital St. Gallen.

Ukraine: A Kuznetsova, Kharkov State Medical University, Kharkov; G Kyselyova, Crimean Republican AIDS centre, Simferopol; M Sluzhynska, Lviv Regional HIV/AIDS Prevention and Control CTR, Lviv.

United Kingdom: (B Gazzard), St. Stephen's Clinic, Chelsea and Westminster Hospital, London; AM Johnson, E Simons, S Edwards, Mortimer Market Centre, London; A Phillips, MA Johnson, A Mocroft, Royal Free and University College Medical School, London (Royal Free Campus); C Orkin, Royal London Hospital, London; J Weber, G Scullard, Imperial College School of Medicine at St. Mary's, London; A Clarke, Royal Sussex County Hospital, Brighton; C Leen, Western General Hospital, Edinburgh.

The following centers have previously contributed data to EuroSIDA:

Infectious Diseases Hospital, Sofia, Bulgaria

Hôpital de la Croix Rousse, Lyon, France

Hôpital de la Pitié-Salpétière, Paris, France

Unité INSERM, Bordeaux, France

Hôpital Edouard Herriot, Lyon, France

Bernhard Nocht Institut für Tropenmedizin, Hamburg, Germany

1st I.K.A Hospital of Athens, Athens, Greece

Ospedale Riuniti, Divisione Malattie Infettive, Bergamo, Italy

Ospedale di Bolzano, Divisione Malattie Infettive, Bolzano, Italy

Ospedale Cotugno, III Divisione Malattie Infettive, Napoli, Italy

Dérer Hospital, Bratislava, Slovakia

Hospital Carlos III, Departamento de Enfermedades Infecciosas, Madrid, Spain

Kiev Centre for AIDS, Kiev, Ukraine

Luhansk State Medical University, Luhansk, Ukraine

Odessa Region AIDS Center, Odessa, Ukraine

HivBivus (Sweden):

Central coordination: L. Morfeldt, G. Thulin, A. Sundström.

Participating physicians (city): B. Åkerlund (Huddinge); K. Koppel, A. Karlsson (Stockholm); L. Flamholc, C. Håkangård (Malmö).

The ICONA Foundation (Italy):

BOARD OF DIRECTORS

A d’Arminio Monforte (President), A Antinori, A Castagna, F Castelli, R Cauda, G Di Perri, M Galli, R Iardino, G Ippolito, GC Marchetti, CF Perno, F von Schloesser, P Viale

SCIENTIFIC SECRETARY

A d’Arminio Monforte, A Antinori, A Castagna, F Ceccherini-Silberstein, A Cozzi-Lepri, E Girardi, S Lo Caputo, C Mussini, M Puoti

STEERING COMMITTEE

M Andreoni, A Ammassari, A Antinori, C Balotta, A Bandera, P Bonfanti, S Bonora, M Borderi, A Calcagno, L Calza, MR Capobianchi, A Castagna, F Ceccherini-Silberstein, A Cingolani, P Cinque, A Cozzi-Lepri, A d’Arminio Monforte, A De Luca, A Di Biagio, E Girardi, N Gianotti, A Gori, G Guaraldi, G Lapadula, M Lichtner, S Lo Caputo, G Madeddu, F Maggiolo, G Marchetti, S Marcotullio, L Monno, C Mussini, S Nozza, M Puoti, E Quiros Roldan, R Rossotti, S Rusconi, MM Santoro, A Saracino, M Zaccarelli.

STATISTICAL AND MONITORING TEAM

A Cozzi-Lepri, I Fanti, L Galli, P Lorenzini, A Rodano, M Shanyinde, A Tavelli

BIOLOGICAL BANK INMI

F Carletti, S Carrara, A Di Caro, S Graziano, F Petrone, G Prota, S Quartu, S Truffa

PARTICIPATING PHYSICIANS AND CENTERS

Italy A Giacometti, A Costantini, V Barocci (Ancona); G Angarano, L Monno, C Santoro (Bari); F Maggiolo, C Suardi (Bergamo); P Viale, V Donati, G Verucchi (Bologna); F Castelli, C Minardi, E Quiros Roldan (Brescia); T Quirino, C Abeli (Busto Arsizio); PE Manconi, P Piano (Cagliari); B Cacopardo, B Celesia (Catania); J Vecchiet, K Falasca (Chieti); A Pan, S Lorenzotti (Cremona); L Sighinolfi, D Segala (Ferrara); F Mazzotta, F Vichi (Firenze); G Cassola, C Viscoli, A Alessandrini, N Bobbio, G Mazzarello (Genova); C Mastroianni, V Belvisi (Latina); P Bonfanti, I Caramma (Lecco); A Chiodera, P Milini (Macerata); A d’Arminio Monforte, M Galli, A Lazzarin, G Rizzardini, M Puoti, A Castagna, G Marchetti, MC Moioli, R Piolini, AL Ridolfo, S Salpietro, C Tincati, (Milano); C Mussini, C Puzzolante (Modena); A Gori, G Lapadula (Monza); N Abrescia, A Chirianni, G Borgia, R Orlando, G Bonadies, F Di Martino, I Gentile, L Maddaloni (Napoli); AM Cattelan, S Marinello (Padova); A Cascio, C Colomba (Palermo); F Baldelli, E Schiaroli (Perugia); G Parruti, F Sozio (Pescara); G Magnani, MA Ursitti (Reggio Emilia); M Andreoni, A Antinori, R Cauda, A Cristaudo, V Vullo, R Acinapura, G Baldin, M Capozzi, S Cicalini, A Cingolani, L Fontanelli Sulekova, G Iaiani, A Latini, I Mastrorosa, MM Plazzi, S Savinelli, A Vergori (Roma); M Cecchetto, F Viviani (Rovigo); G Madeddu, P Bagella (Sassari); A De Luca, B Rossetti (Siena); A Franco, R Fontana Del Vecchio (Siracusa); D Francisci, C Di Giuli (Terni); P Caramello, G Di Perri, S Bonora, GC Orofino, M Sciandra (Torino); M Bassetti, A Londero (Udine); G Pellizzer, V Manfrin (Vicenza) G Starnini, A Ialungo(Viterbo).

Nice HIV Cohort (France):

Central coordination: C. Pradier*, E. Fontas, K. Dollet, C. Caissotti.

Participating physicians: P. Dellamonica, E. Bernard, J. Courjon, E. Cua, F. De Salvador-Guillouet, J.Durant, C. Etienne, S. Ferrando, V. Mondain-Miton, A. Naqvi, I. Perbost,S. Pillet , B. Prouvost-Keller, P. Pugliese, V. Rio, K. Risso, P.M. Roger.

SHCS (Swiss HIV Cohort Study, Switzerland):

The data are gathered by the Five Swiss University Hospitals, two Cantonal Hospitals,15 affiliated hospitals and 36 private physicians (listed in http://www.shcs.ch/180-health-care-providers).

Members of the Swiss HIV Cohort Study :

Aubert V, Battegay M, Bernasconi E, Böni J, Braun DL, Bucher HC, Calmy A, Cavassini M, Ciuffi A, Dollenmaier G, Egger M, Elzi L, Fehr J, Fellay J, Furrer H (Chairman of the Clinical and Laboratory Committee), Fux CA, Günthard HF (President of the SHCS), Haerry D (deputy of "Positive Council"), Hasse B, Hirsch HH, Hoffmann M, Hösli I, Kahlert C, Kaiser L, Keiser O, Klimkait T, Kouyos RD, Kovari H, Ledergerber B, Martinetti G, Martinez de Tejada B, Marzolini C, Metzner KJ, Müller N, Nicca D, Pantaleo G, Paioni P, Rauch A (Chairman of the Scientific Board), Rudin C (Chairman of the Mother & Child Substudy), Scherrer AU (Head of Data Centre), Schmid P, Speck R, Stöckle M, Tarr P, Trkola A, Vernazza P, Wandeler G, Weber R*, Yerly S.

Financial acknowledgements:

Data on Adverse Events (D:A:D) Study:The D:A:D study was supported by the Highly Active Antiretroviral Therapy Oversight Committee (HAARTOC), a collaborative committee with representation from academic institutions, the European Agency for the Evaluation of Medicinal Products, the United States Food and Drug Administration, the patient community, and pharmaceutical companies with licensed anti-HIV drugs in the European Union: AbbVie, Bristol-Myers Squibb, Gilead Sciences Inc., ViiV Healthcare, Merck & Co Inc. and Janssen Pharmaceuticals. Supported also by a grant [grant number DNRF126] from the Danish National Research Foundation (CHIP & PERSIMUNE); by a grant from the Dutch Ministry of Health, Welfare and Sport through the Center for Infectious Disease Control of the National Institute for Public Health and the Environment to Stiching HIV Monitoring (ATHENA); by a grant from the Agence nationale de recherches sur le sida et les hépatites virales [ANRS, Action Coordonnée no.7, Cohortes] to the Aquitaine Cohort; The Australian HIV Observational Database (AHOD) is funded as part of the Asia Pacific HIV Observational Database, a program of The Foundation for AIDS Research, amfAR, and is supported in part by a grant from the U.S. National Institutes of Health’s National Institute of Allergy and Infectious Diseases (NIAID) [grant number U01-AI069907] and by unconditional grants from Merck Sharp & Dohme; Gilead Sciences; Bristol-Myers Squibb; Boehringer Ingelheim; Janssen-Cilag; ViiV Healthcare. The Kirby Institute is funded by The Australian Government Department of Health and Ageing, and is affiliated with the Faculty of Medicine, The University of New South Wales; by grants from the Fondo de Investigación Sanitaria [grant number FIS 99/0887] and Fundación para la Investigación y la Prevención del SIDA en Espanã [grant number FIPSE 3171/00], to the Barcelona Antiretroviral Surveillance Study (BASS); by the National Institute of Allergy and Infectious Diseases, National Institutes of Health [grants number 5U01AI042170-10, 5U01AI046362-03], to the Terry Beirn Community Programs for Clinical Research on AIDS (CPCRA); by primary funding provided by the European Union’s Seventh Framework Programme for research, technological development and demonstration under EuroCoord grant agreement n˚ 260694 and unrestricted grants by Bristol-Myers Squibb, Janssen R&D, Merck and Co. Inc., Pfizer Inc., GlaxoSmithKline LLC, (the participation of centres from Switzerland is supported by The Swiss National Science Foundation (Grant 108787)) to the EuroSIDA study; by unrestricted educational grants of AbbVie, Bristol-Myers Squibb, Gilead Sciences, GlaxoSmithKline, Pfizer, Janssen Pharmaceuticals to the Italian Cohort Naive to Antiretrovirals (The ICONA Foundation); and financed within the framework of the Swiss HIV Cohort Study, supported by the Swiss National Science Foundation (grant #148522) and by the SHCS research foundation.

The content of this publication is solely the responsibility of the authors and does not necessarily represent the official views of any of the institutions mentioned above.
