# Supplementary material for: Cardiovascular disease (CVD) and chronic kidney disease (CKD) event rates in HIV-positive persons at high predicted CVD and CKD risk: A prospective analysis of the D:A:D observational study
Source: PLoS Med. 2017 Nov 7;14(11):e1002424. doi: 10.1371/journal.pmed.1002424 (PMC5675358; doi:10.1371/journal.pmed.1002424)
Supplement: S2 Text — (DOC) [file pmed.1002424.s005.doc]

**STUDY CONCEPT SHEET**

**An analysis of the overlap of Individuals with both high D:A:D CVD risk and renal risk score and options to optimise their ART**

| **Proposed by** | Mark Boyd, Matthew Law  Kirby Institute, UNSW Australia |
| --- | --- |
| **Study rationale** | With the success of ART extending the life expectancy of PLH and an increasing proportion of PLH reaching middle-age, there is concern about the extent to which they will develop conditions which may limit their health, wellbeing and longevity. There is evidence that a number of comorbidities are more prevalent in PLH. This may be as a result of particular lifestyle behavioural factors more common in PLH and/or may be attributable to HIV itself, even in those who have gained and maintained optimal virological control on ART.  As a result there has been interest in developing disease prediction models and risk scores to help clinicians and patients assess relative risk of the future development of comorbidities and adverse health outcomes and intervene appropriately. The D:A:D study was established to assess whether the use of ART is associated with an increased risk of CVD^1^. More recently the cohort data has been used to develop a validated risk score for the development of at least moderate chronic kidney disease (CKD; defined as first eGFR <60 ml/min/1.72m^2^)^2^. The understanding of how to administer ART is strongly evidence-based. However, this knowledge is almost entirely limited to healthy PLH with low to moderate CVD risk and relatively normal renal function (the majority of RCTs are performed in PLH with a screening eGFR >60 ml/min/1.72 m^2^). The D:A:D study has revealed various associations between specific ARVs and adverse CVD risk and renal outcomes^3,4^.  It is unknown whether PLH with both a high CVD score and an at least moderate renal risk score are at even greater risk of unfavourable outcomes and should therefore be managed in a more intensive or otherwise different manner. |
| **Hypothesis** | People living with HIV with risk factors for both elevated CVD and renal risk are at greater than additive risk for subsequent severe CVD and renal endpoints. |
| **Study objectives** | This study proposal has two main aims:  *Primary*   - Describe the proportion of D:A:D participants with a high predicted risk for CVD and CKD and whether their risk of severe CVD and renal endpoints is greater than would be expected from an additive effect alone.   *Secondary objectives:*   - Describe the demographic and clinical characteristics of the cohort of patients with the combined CVD/renal risk compared with those without these risks - Assess and describe the extent to which ART might be modified to minimise future CVD and renal risk. |
| **Inclusion criteria** | Those eligible will be participants with all variables required to calculate both CVD risk and CKD risk entered into the D:A:D database (with eligibility defined as eligibility from that time point on with at least one follow-up visit thereafter).  Since D:A:D was originally established to look for associations between ART and CVD, the collection of variables associated with renal disease lags the time at which variables associated with CVD was collected. Therefore the baseline for the study will be defined as that timepoint at which all variables for an assessment of both CVD and renal scores exists.  Participants with previous CVD or kidney events will be excluded. |
| **Statistical plan** | ***Primary objective***  Demographic characteristics, current virological and immunological status and current and past ART will be summarised at first follow-up with complete covariate data.  CVD and CKD risk will be predicted using the D:A:D calculators at start of analysis follow-up, and patients stratified into risk groups as:  5-year CVD risk <1%, 1-5%, 5-10% and >10%  CKD risk score low (0), medium (1-4) and high (5+)  CVD and renal event rates will be calculated by cross-strata of predicted risk.  ***Secondary objective***  CVD and CKD predicted risk will also be calculated at the patient’s most recent visit within the last two years (since February 2014).  Demographic characteristics, current virological and immunological status and current and past ART will be summarised by predicted CVD and CKD risk.  Availability of safer ART agents will be classified as to number available (1, 2 or ≥3 ARVs). A similar strategy was employed in the RATE study^5^. |
| **Timeline:** | Aim for CROI 2017 abstract and publication same year. |
| **References:** | 1 Friis-Moller N, et al. An updated prediction model of the global risk of CVD in HIV+ persons: the D:A:D:study. Eur J Prev Cardiol 2015.  2 Mocroft A, et al. Development and validation of a risk score for CKD in HIV infection using prospective cohort data. PLoS Med 12(3): e1001809.  3 Friis-Møller N, et al. [Combination antiretroviral therapy and the risk of myocardial infarction.](http://www.ncbi.nlm.nih.gov/pubmed/14627784) N Engl J Med. 2003 Nov 20;349(21):1993-2003.  4 Ryom L, et al. [Association between antiretroviral exposure and renal impairment among HIV-positive persons with normal baseline renal function: the D:A:D study.](http://www.ncbi.nlm.nih.gov/pubmed/23382571) J Infect Dis. 2013 May 1;207(9):1359-69  5 Achhra AC,et al. [Moving away from Ritonavir, Abacavir, Tenofovir, and Efavirenz (RATE)--agents that concern prescribers and patients: a feasibility study and call for a trial.](http://www.ncbi.nlm.nih.gov/pubmed/24968324) PLoS One. 2014 Jun 26;9(6):e99530. |
